# Supplementary material for: Identification of DDX60 as a Regulator of MHC-I Class Molecules in Colorectal Cancer
Source: Biomedicines. 2022 Dec 1;10(12):3092. doi: 10.3390/biomedicines10123092 (PMC9775109; doi:10.3390/biomedicines10123092)
Supplement: Supplementary file 1 [file biomedicines-10-03092-s001.zip › biomedicines-1950486-supplementary.pdf]

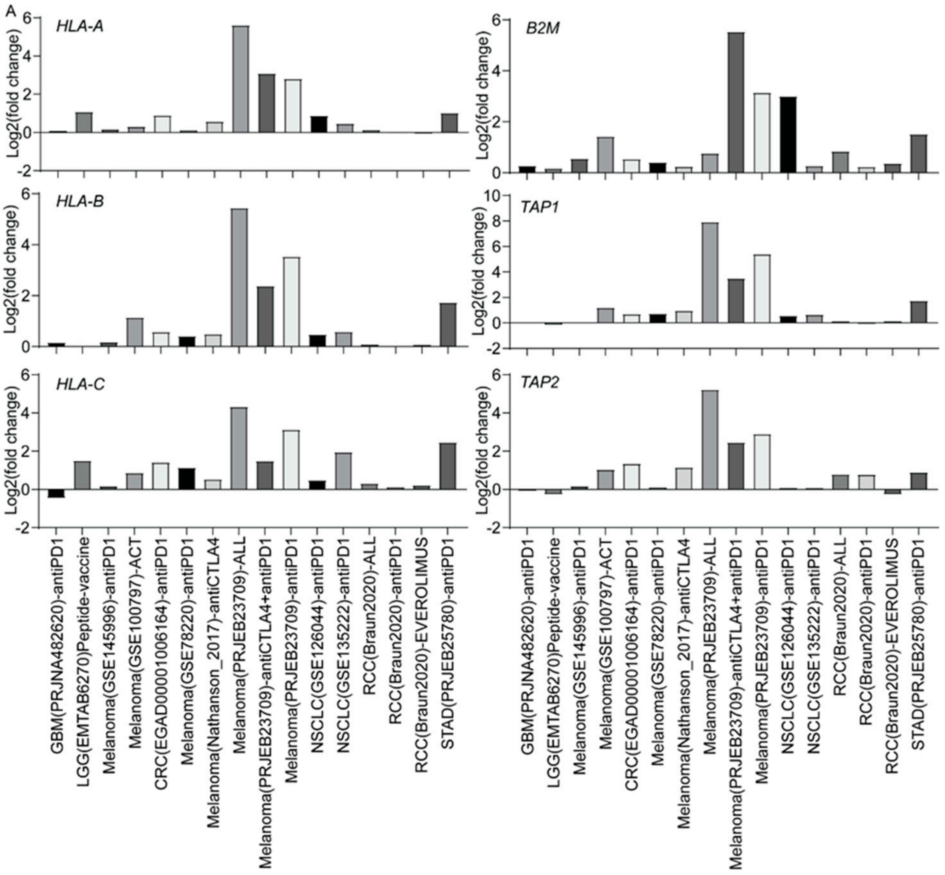


**Figure S1**. **MHC-I expression is correlated to responders in immunotherapy**. The mRNA expression levels (fold change of Log2) of MHC-I molecules in the tumors from the immunotherapy responders and non-responders in different solid cancer. The gene expression data were obtained from Tumor Immunotherapy Gene Expression Resource (TIGER) at http://tiger.canceromics.org/#/home. The numbers in parentheses are the original research data storage codes. GBM: Glioblastoma; LGG: Low-grade gliomas; NSCLC: Non-small cell lung cancer; RCC: Renal cell cancer; STAD: Stomach adenocarcinoma.

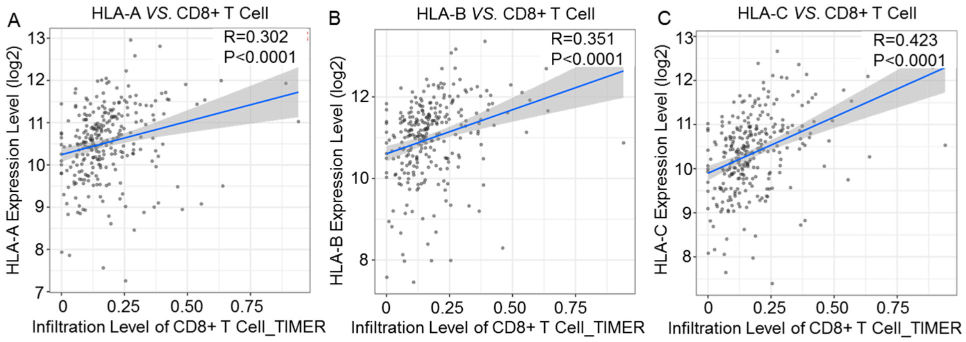


**Figure S2. The expression of MHC-I molecules correlates with CD8+ T cell infiltration in CRC.** (A-C) The diagrams plot the regression correlation of CD8 T cell infiltration and the expression of MHC-I molecules in CRC. The infiltration level of CD8+ T cell was given by TIMER2.0 analysis of TCGA colorectal cancer patient datasets (Taiwen Li et. al., Nucleic Acids Research, 2020). The HLA-A/B/C mRNA expression were obtained from cBioportal (https://www.cbioportal.org/). The partial Spearman's correlation was used to perform the association analysis. R: spearman's rho. The same patientshave been analyzed for both MHC-I expression and CD8+ T cell infiltration. The figures concern TILs.

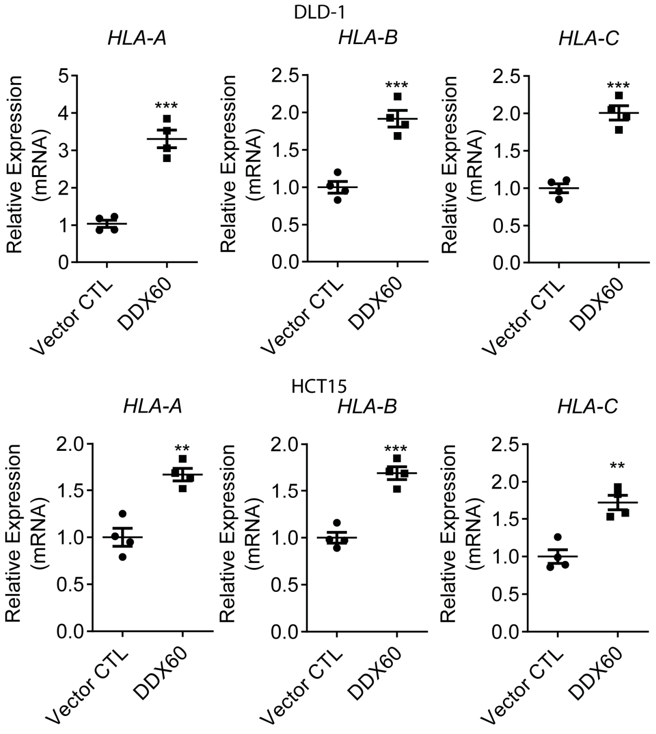


**Figure S3. DDX60 regulated the expression of MHC-I molecules in CRC cells.** RT-PCR results showed the expression of *HLA-A/B/C* in vector control or DDX60-overexpressed DLD-1 and HCT-15 cells. Beta-actin was used as internal control.

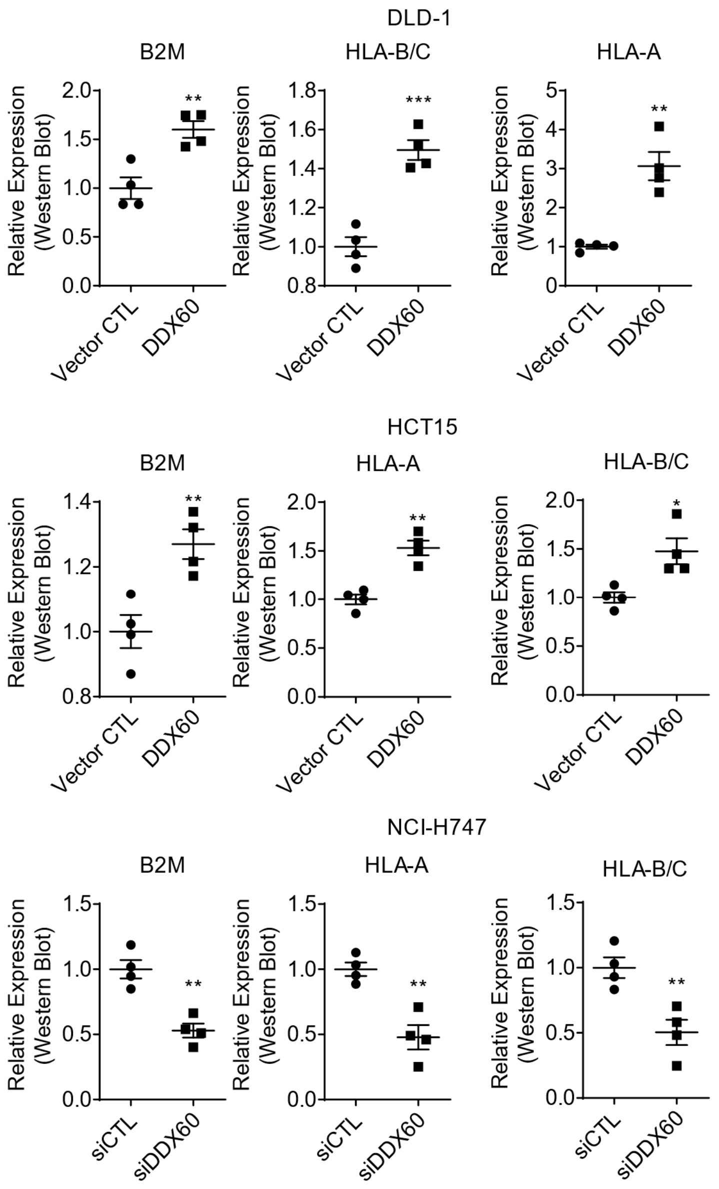


**Figure S4. DDX60 regulated the expression of MHC-I molecules in CRC cells.** Western Blot quantification analysis results showed the expression of B2M, HLA-A/B/C in control DDX60-overexpressed, or DDX60-Knockdown cells from Figure 4A, 4B, and 4G. Beta-actin was used as internal control.

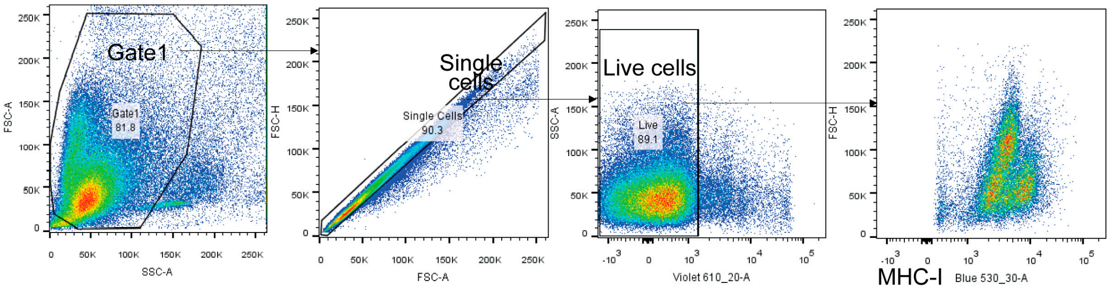


**Figure S5. Gating strategies for MHC-I level analysis by FACS.**
